# Genetic structure and common ancestry expose the dingo-dog hybrid myth

**DOI:** 10.1093/evlett/qrae057

**Published:** 2024-10-19

**Authors:** Andrew R Weeks, Peter Kriesner, Nenad Bartonicek, Anthony van Rooyen, Kylie M Cairns, Collin W Ahrens

**Affiliations:** Cesar Australia, Brunswick, Australia; School of BioSciences, The University of Melbourne, Parkville, Australia; Cesar Australia, Brunswick, Australia; Cesar Australia, Brunswick, Australia; Cesar Australia, Brunswick, Australia; Centre for Ecosystem Science, Evolution & Ecology Research Centre, School of Biological, Earth and Environmental Sciences, University of New South Wales, Sydney, Australia; Cesar Australia, Brunswick, Australia

**Keywords:** dingo, canids, evolution, introgression, hybridization

## Abstract

The evolutionary history of canids has been shown to be complex, with hybridization and domestication confounding our understanding of speciation among various canid lineages. The dingo is a recent canid lineage that was completely isolated from other canids for over 5000 years on the Australian mainland, but the introduction of domestic dogs in 1788 has placed doubt on its independence, with recent studies highlighting hybridization between dingoes and domestic dogs. Using genomic single nucleotide polymorphism data from 434 Australian canid samples, we explicitly test for introgression between closely related canid groups and dingoes. We found no evidence of introgression between dingoes and domestic dogs and show that previous work has likely mischaracterized shared ancestral genetic variation as evidence for hybridization. Further, New Guinea Singing Dogs are the only canid group that significantly shared genetic variation with dingoes, which fits with our understanding of previous phylogenetic analyses. Despite more recent sympatric distributions with dogs, dingoes have likely maintained their independence since their arrival in Australia, even in areas with high lethal control, indicating that their evolutionary trajectory is currently being conserved. The future conservation of the dingo lineage will require policies that promote coexistence pathways between humans and dingoes that protect rangeland systems and the dingoes’ evolutionary future.

## Introduction

Canids are an important group of animals from ecological and social perspectives, but also represent a unique evolutionary history, providing insight into biological processes around speciation and hybridization (Gopalakrishnan et al., 2018; Perini et al., 2010; Porto et al., 2023; Slater, 2015). The dog split from their wild wolf relatives at least 15,000 years ago, although the exact date of divergence and domestication is unknown (Shannon et al., 2015). Hybridization between dogs and wild canids is relatively common in the United States and Europe and has been identified in Australia (Galaverni et al., 2017; Gopalakrishnan et al., 2018; Stephens et al., 2015). The New Guinea Singing Dog (NGSD) and the Australian dingo are both ancient canid lineages which are thought to have arisen from south-east Asian dogs (Zhang et al., 2020), although their domestication status is unclear. Understanding the evolutionary history of canid radiations is important for disentangling the role of hybridization in the speciation process, but it also provides insight into the impact domestication may have on speciation.

The Australian dingo is a wild canid that arrived in Australia approximately 5,000–8,300 years ago based on molecular analyses (Ballard & Wilson, 2019; Souilmi et al., 2024; Zhang et al., 2020) with an unknown domestication history (Ballard & Wilson, 2019). Genomic evidence indicates that, like the NGSD, they are a basal lineage to many domestic dogs (Ballard et al., 2023; Field et al., 2022). However, other analyses show that dingoes are grouped within the Asian and Arctic domestic dog clade (Surbakti et al., 2020), suggesting that dingoes are an early offshoot of the domestic dog group. Dingoes have retained behaviors similar to other wild canids, such as wolves and coyotes, forming packs with a social hierarchy often consisting of a dominant female and male (Corbett, 1995; Glen et al., 2007) and hunting large prey (e.g., kangaroos and wallabies) either solo or in small groups (Pollock et al., 2022). Despite behavioral differences, dingoes can hybridize with domestic dogs (Ballard & Wilson, 2019), a characteristic common to many canid species, including wolves, coyotes and golden jackals (Galaverni et al., 2017). In fact, genomics has revealed complex evolutionary patterns of ongoing hybridization between wolves and domestic dogs, particularly in regions where their distributions overlap (Fan et al., 2016; Galaverni et al., 2017; Pilot et al., 2018). However, unlike wolves, dingoes were isolated from other canid groups for over 5,000 years, with domestic dogs only introduced to Australia in 1788 (Field et al., 2022). The dingo’s complex evolutionary history makes it the perfect group for disentangling the influence of domestication, social behaviors and hybridization on speciation and evolution.

Genetic research has provided contrasting evidence for hybridization between dingoes and domestic dogs in Australia. Stephens et al. (2015) suggested that dingoes hybridize readily with domestic dogs, particularly in southeastern Australia, which was leading to the decline of “pure” dingoes. This narrative has been reiterated in many recent and past studies (Corbett, 2001; Elledge et al., 2006; Field et al., 2022; Glen et al., 2007; Savolainen et al., 2004; Stephens et al., 2022; Zhang et al., 2020), and led to a difficult political landscape in southeastern Australia where “pure” dingoes are protected, while dingo-dog hybrids are legally killed to protect livestock (Victorian Government, 2023a). However, a more recent study using genomic single nucleotide polymorphisms (SNPs) found that this hybridization was mostly mischaracterized (Cairns et al., 2023) and places substantial doubt around the frequency of hybridization between dingoes and domestic dogs. Collectively, these studies have relied upon STRUCTURE analyses to identify recent admixture between dingoes and domestic dogs. Yet STRUCTURE can be prone to overinterpretation and misuse (Novembre, 2016). For instance, (a) STRUCTURE cannot differentiate between incomplete lineage sorting (ILS) and admixture (Durand et al., 2011), (b) inadequate correction for patterns of isolation-by-distance (IBD) can lead to wrong cluster assignments (Perez et al., 2018), (c) it assumes no inbreeding, which can lead to inferences of a high proportion of null alleles (Falush et al., 2007), and (d) it cannot account for biological processes that deviate from Hardy-Weinberg equilibrium (HWE) (Pritchard et al., 2000), such as bottlenecks. Given the relatively recent split of the dingo lineage from other canids, ILS and common ancestry are highly likely (Lopes et al., 2021), and dingo social structure predisposes them to inbreeding (Wallach et al., 2009). While STRUCTURE can be an important method for identifying admixture under the right circumstances, in the case of dingoes, other methods are likely to be more informative and less erroneous.

Accurately characterizing hybridization and introgression at the population level is a first step to understanding the continued evolution of dingoes in Australia as a separate lineage of wild canid. Here, we have used genomic SNPs and a large sample size of free ranging canids from southeastern Australia to show that the Australian dingo has maintained independence from domestic dogs throughout its range, with no evidence of effective hybridization (measurable introgression of contemporary dog specific genetic variation into free ranging dingo populations), a finding that is in stark contrast to decades of previous research. Our results show that dingoes still remain on a path towards speciation and represent a unique evolutionary process whereby a potentially domesticated animal is on a trajectory towards becoming a new species.

## Methods

### Samples

A total of 434 individual Australian canid samples (tissue or extracted DNA) were supplied for genetic analysis through various sources from locations around Australia (Supplementary Table 1). The majority of the Victorian samples were collected by the Victorian Government as part of the Victorian Wild Dog Program (2014–2019), with most samples collected from northeast Victoria (Alpine population) and western Victoria (Mallee population). Other samples were supplied to the authors by various sources, including government officers, private landholders, conservation organizations and dog owners.

In total, the samples comprised 380 free ranging field-collected individuals, which were morphologically identified as dingoes or potential hybrids (between domestic dogs and dingoes). The remaining 54 samples were from domestic and captive individuals from Australia comprising 39 domestic dogs, seven known hybrids born in captivity (F1-dingo × dog crosses), and eight known backcrossed individuals born in captivity (dingo-dog hybrid × dingo crosses).

**Figure 1.**
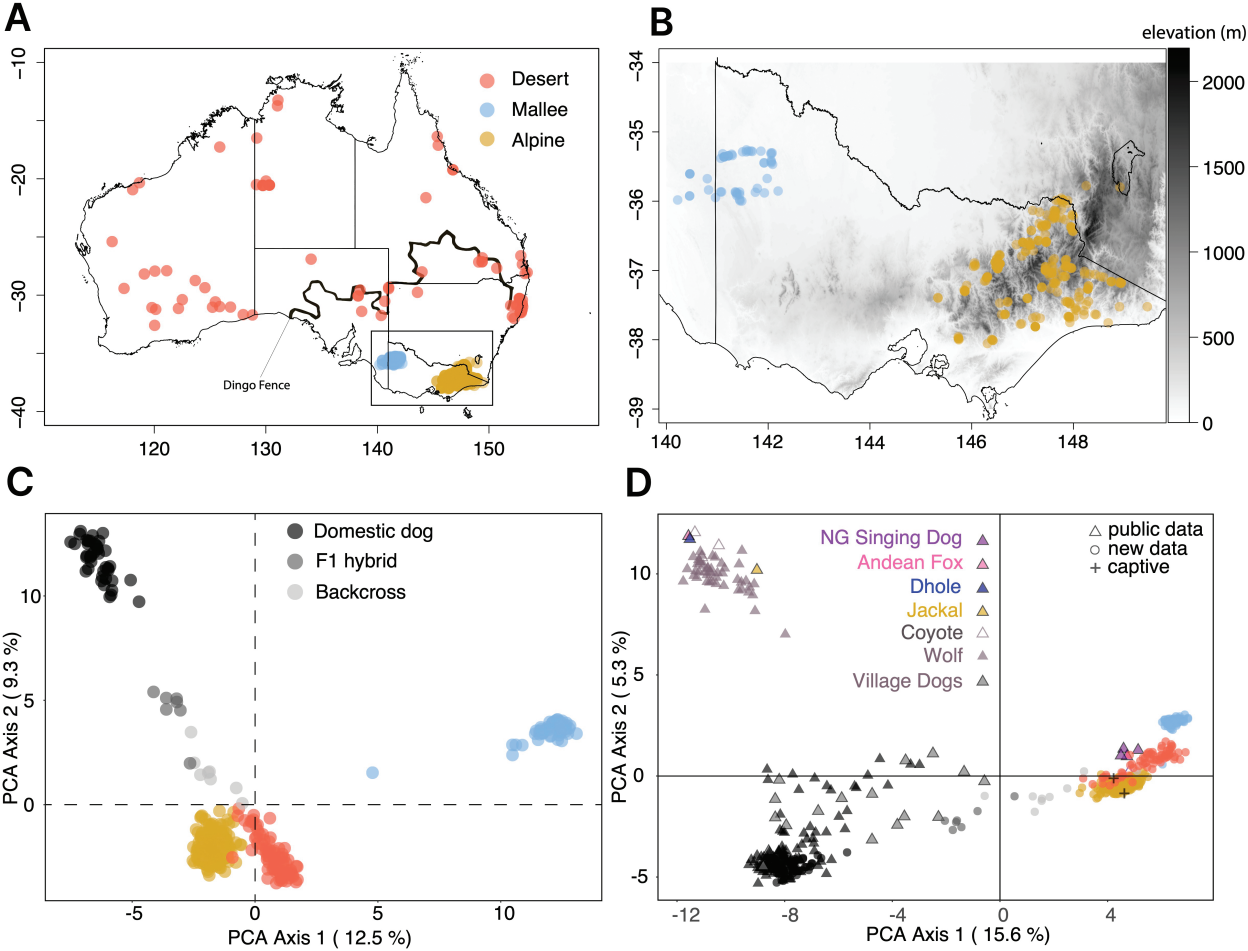
Sample location information across Australia (A) and in southeastern Australia (B), and two principal components analyses (C) and (D). PCAs show contextualization with known domestic and hybrid dogs for the Australian dataset (C) and for the global dataset (with the color and gray-scale points from A, B, and C) (D). The black line in (A) is the 5,614 km long dingo fence that hypothetically limits dingo movement into the southeast region.

The free ranging individuals were sampled at a wide range of separate locations across mainland Australia (Figure 1A), but the majority were collected from southeastern Australia, where previous research had indicated a high degree of hybridization (Figure 1B). The collection locations approximately align with either of two dingo ecotypes (“Alpine” or “Desert”) proposed by Ballard & Wilson (2019), but also include individuals from a third isolated population that persists west of the Alpine population, called the “Mallee” population (Cairns et al., 2023). We use these designations for describing dingo/free ranging canid samples in Australia henceforth.

### DNA extraction and genomic SNP genotyping

All tissue samples were sent to Cesar Australia and stored in ethanol (>90%). Genomic DNA was extracted from individual samples using Qiagen blood and tissue kits following the manufacturer’s instructions on a QIAcube robot (Qiagen). The concentration of DNA for each sample was measured using a Qubit fluorometer and standardized (between 10 and 20 ng/ml) prior to being forwarded at 4 °C to Diversity Arrays Pty Ltd (www.diversityarrays.com) for subsequent reduced representation DNA sequencing (ddRAD) using the DArTSeq methodology (Kilian et al., 2012). A total of 29 technical replicates were included in the analyses to ensure data quality within and between plates of samples submitted for sequencing.

### Bioinformatic analyses

Bioinformatic pipelines were performed for two datasets representing (a) new data from this study (434 genotypes; Australia dataset) and (b) this data in context with publicly available canid SNP data (634 genotypes; global dataset). The Australian dataset (Supplementary Table S1) focused on population structure of dingoes alone and relationship between dingo-dog hybrids with known history, while the global dataset aimed to assess signatures of admixture between dingoes and multiple canid species and hybrids and to uncover patterns of evolutionary history between broader groups of canids and dingoes. The latter dataset provides context regarding any relationships or sharing found between dingoes and dogs. This broader canid group included samples with 91 million SNPs and small indels across 722 canids (Plassais et al., 2019), of which we kept 200 samples for analyses. These additional canid genotypes included one Andean fox, one dhole, three coyotes, one golden jackal, and 48 wolves as outgroups, along with one dingo, four NGSD, 22 “village” dogs (dogs that are free ranging but associated with human settlements), and 119 domestic dogs (Supplementary Table S2). The domestic dog genotypes chosen represent over 100 different recognized dog breeds, including three Australian cattle dogs and some other breeds popular with Australian farmers. Raw reads for the Australian dataset were demultiplexed with process_radtags v.2.55 (PstI enzyme sites) from the Stacks package (Catchen et al., 2013), where it also discarded low quality reads (Phred < 10) and then mapped to the CanFam3.1 genome (GCF_000002285.3_CanFam3.1_genomic.fna) using bwa v.0.7.17-r1188 (Li & Durbin, 2009). We also called variants for the Australian dataset mapped against the novel dingo genome ASM325472v2 (Field et al., 2022) to check for any biases. The mapping genome yielded few differences (e.g., Supplementary Figures S1 and S2); we therefore report results from the dog genome (CanFam3.1) for dataset consistency throughout the paper. The Gstacks v.2.55 module from the Stacks package was run with default parameters on all unique samples and a number of additional data duplicates to evaluate overall error rate. The Stacks “Populations” module was then run with -r 0.7 (minimum percentage of individuals in a population required to process a locus for that population). Chromosomes output from Stacks were reannotated with bcftools v.1.9 (Li, 2011) to chromosomes 1–38 and only the first 38 chromosomes were used in the analysis (the sex chromosome (39) was removed). Indels were removed using bcftools. The global dataset was mapped to the CanFam3.1 genome using bwa v.0.7.17-r1188. To obtain the global dataset, the intersection of the resulting Australian and other canid vcf files was found with bcftools isec (v.1.9), and the resulting overlaps were merged. The resulting VCF files were filtered for missing data (0.8), minor allele frequency (0.03), and linkage disequilibrium (*r*^2^ = 0.7) using bcftools.

### Population genetic analyses

Basic population genetic statistics were evaluated using the dartR (Gruber et al., 2018; Mijangos et al., 2022) and heirfstat v0.5-11 (Goudet & Jombart, 2015) packages in R. Observed (*H*_O_), expected (*H*_E_), unbiased heterozygosity (u*H*_E_), and inbreeding coefficient (*F*_IS_) were calculated using the gl.report.heterozygosity function. *F*_IS_ values were considered significant if the 95% confidence intervals estimated from bootstrapping using boot.ppfis in the hierfstat package did not overlap zero. Global and pairwise population differentiation (*F*_ST_) was estimated using the gl.fst.pop function in dartR. Subsequent analysis to determine population structure and relationships between canid groups was undertaken with principal component analyses (PCA), Discriminant Analysis of Principal Components (DAPC), and STRUCTURE (Pritchard et al., 2000). PCA was performed using the gl.pcoa and gl.pcoa.plot functions in dartR package, and DAPC using the dapc function in adegenet (Jombart, 2008; Jombart & Ahmed, 2011). DAPC is a multivariate method designed to identify and describe clusters of genetically related individuals that does not assume a particular population genetics model and is free of assumptions on HWE and linkage equilibrium (Jombart et al., 2010). We used this method for re-analyzing the original microsatellite dataset (doi:10.5061/dryad.2rd32) from Stephens et al. (2015).

We recognize that STRUCTURE is an inappropriate method to estimate admixture in this canid complex, but we perform the STRUCTURE analyses to show that our data is similar to data from previous work. Individual assignment analysis was undertaken using STRUCTURE v.2.3.4 through structure_threader (Pina-Martins et al., 2017). STRUCTURE uses a Bayesian clustering approach applying Markov Chain Monte Carlo (MCMC) estimation and enables membership probabilities of individuals to populations or clusters when there is assumed admixture (Porras-Hurtado et al., 2013). Parameter optimization was performed with a burnin period of 50,000 iterations and MCMC iterations of 250,000 for values of *K* = 1–10 and 10 replicates per *K* using the admixture and correlated allele frequencies models. The ANCESTDIST flag was used to calculate the Bayesian probability intervals around the *q*-matrix and provide confidence around the ancestral clusters. The optimum *K*-value was determined using the Evanno method (Evanno et al., 2005).

To further test assumptions of population structure, we used a multivariate method (sPCA) to estimate significance of IBD as a driver of population structure. To compute spatial autocorrelation, we used the spca function in the adegenet package and significance of spatial autocorrelation was ascertained by using the global.rtest function with 1,000 permutations. We tested for IBD on our own SNP dataset, and also the microsatellite dataset from Stephens et al. (2015).

We found a significant inbreeding coefficient in all three broadly grouped populations (Alpine, Desert, Mallee), but one population (Mallee) was nearly double that of the others and was less likely to be due to a Wahlund effect (grouping of subpopulations). We therefore also tested specifically for a bottleneck in this population using DIYABC-rf (Collin et al., 2021). DIYABC-rf integrates simulations under custom evolutionary scenarios and random forest treatments to evaluate the power and accuracy of inferences. Scenarios are evolutionary models that describe the relationship of populations and are user-defined. Both scenario choice (population level phylogeny) and parameter estimation (in our case, effective population size, and population decline due to bottlenecks) are performed comparing simulations and real data. We used DIYABC-rf three times to identify the best scenario for each group (two bottlenecks, one bottleneck, and zero bottlenecks; details in the uploaded header RF files—see data availability statement). In total, we ran 10 scenarios that included two bottlenecks, seven scenarios with one bottleneck, and five scenarios with zero bottlenecks, for a total of 22 scenarios. Then, we took the top scenario from each bottleneck group and ran these three top scenarios together in a fourth DIYABC-rf analysis to identify which scenario best explains the data. The population size priors were uniformly distributed between 10 and 10,000. Time parameters (t1 and t2) were normally distributed (1–1,000 with a mean of 50), and generation time (db) were normally distributed (1–20 with a mean of 10; other means were explored, but these parameters yielded the best data fit as discussed in Collin et al. (2021)). For each of the three top models, we ran each model independently with 2,000 simulations and 1,000 trees to estimate the following parameters, effective population size (*N*_e_) and bottleneck decline (NXb).

### Dingo-dog admixture analyses

To understand hybridization and patterns of gene flow between domestic dogs and dingoes, and their relationship with the dingo’s closest living relative the NGSD, we explored the evolutionary history between these canid groups since their Australian isolation. Using the wolf individuals as the outgroup and dingoes as the target group, we employed D-statistics (ABBA BABA) to tease apart the contribution of introgression despite shared variation due to ILS using the following formula (Martin et al., 2015):


$$\begin{array}{l} D=\;\frac{\sum C_{ABBA}-C_{BABA}}{\sum C_{ABBA}+C_{BABA}} \end{array}$$

where *C*_*ABBA*_ and *C*_*BABA*_ are counts of a particular topology. This can partially answer the question we are interested in: has there been admixture between dingoes and dogs since Australian isolation? The goal of the D-stat analysis is to quantify the amount the discordant genealogies are skewed towards an ABBA or BABA topology. This is calculated based on the frequency of the derived allele and ancestral allele in each species. The interpretation of this D-statistic is that a positive number is from an excess of ABBA topologies and a negative number is from an excess of BABA topologies. Here, we used the R package admixtools v 2.0.4 (Maier et al., 2023) to employ the D-stat method (Martin et al., 2015). The D-stat test was performed using the qpdstat function in admixtools with the f4mode = FALSE. In the first iteration, we estimated the D-stat on the full dingo group, where the dingo group was the target (population one all free ranging canid samples in our dataset; Supplementary Table S1) with NGSD (population 2; four samples from the public repository; Supplementary Table S2), dogs (population 3; 39 domesticated dog samples from our dataset; Supplementary Table S1), and wolves (population 4; outgroup; 33 samples from the public dog repository; Supplementary Table S2), which followed the phylogenetic tree developed using genetic distance. The phylogenetic tree was created using the aboot function in the poppr package (Kamvar et al., 2014) with 1,000 bootstraps on Nei’s distance between populations (Nei, 1972). We ran a second test with the same groupings, flipping populations two, and three to see if the admixture signal was lost when dingo and NGSD groups were on opposite sides. For the second analysis, we split the free ranging dingoes into their three broad populations (Alpine, Desert, Mallee), aiming to test whether some populations might have a higher signal of introgression than others. Specifically, we ran the D-stat test three separate times: (a) Alpine:NGSD-dogs:wolves, (b) Desert:NGSD-dogs:wolves, and (c) Mallee:NGSD-dogs:wolves.

We tested how much genetic variation in the dingo group is from NGSD or dogs using the qpadm function in admixtools to estimate weight and significance of relative admixture contributions from plausible models between left and target groups. Here, we wanted to see how NGSD and dog groups (left) explicitly contributed to contemporary dingo (target) genetic variation. For the outgroups (right), we used the following canid groups from the public dataset: Andean Fox, Dhole, Jackal, Coyote. Model fit was estimated for the full model using a chi square test in a drop population paradigm, where models are compared to one another with all possible combinations of source populations. Here, if the *p*-value is below 0.05, then the model is not a good fit of the data and the model was not further investigated (Harney et al., 2021). If the model is a good fit (*p* >  0.05), each source contributor in the model is given a *p*-value and weight of contribution and a significant *p*-value rejects the null hypothesis (of no admixture), which indicates a significant amount of admixture between dingoes and the source contributor (displayed as barplots). For relative admixture weights, which tell us the proportions contributed to dingoes, all contributions add up to one regardless of the total admixture contributed. For the full dingo dataset (i.e., without populations), we ran three two-lineage models (1. Target: Dingo, Left: wolf & dog; 2. T: dingo, L: 3. wolf & NGSD; T: dingo, L: dog & NGSD) to see which lineage(s) significantly contributed genetic variation to the dingo. We also ran one three-lineage model (T: dingo, L: dog & NGSD & wolf). For the dingo population dataset (alpine, desert, & mallee), we ran the dog & NGSD model for each population separately (T: dingo population, L: dog & NGSD) and we also ran the dog group as the only possible source for the Alpine population (T: dingo-alpine, L: dog) and added the NGSD group to the reference population because the Alpine population has previously been identified as having the highest proportion of dog admixture (Stephens et al., 2015).

We also used Treemix (Pickrell & Pritchard, 2012) as an independent method to estimate admixture events between canid groups. Treemix uses population level allele frequencies to create a maximum likelihood tree and infer admixture events. For this analysis, we used the global dataset with all canid groups but also used a more focal dataset that included dingo populations, NGSD, dogs, wolves, hybrids, and backcrosses (Supplementary Table S1). For both analyses, we ran Treemix for 0–5 inferred admixture events with the public wolf samples as the outgroup and bootstrapped 1,000 times. The optimal number of admixture events was determined by comparing residuals of each analysis using the supplied R code (https://github.com/carolindahms/TreeMix).

## Results

### Dingo population structure

We identified 2,466 SNPs for the Australian dataset (dingoes, backcrosses, hybrids, and dogs) and 5,422 SNPs for the global dataset (the Australian dataset plus public data consisting of dogs, wolves, NGSD, jackals, Andean foxes, coyotes, d’hole). We split the dingo samples into three broad categories (Desert, Mallee, and Alpine) as defined by Ballard & Wilson (2019) for Desert and Alpine ecotypes, and Mallee more recently by Cairns et al. (2023). These groups show moderate differentiation among one another (*F*_ST_ = 0.18), with pairwise *F*_ST_ estimates ranging from 0.08 to 0.42 (Table 1), along with different levels of inbreeding and heterozygosities (Table 2). Given the broad geographic regions covered by our samples, there is likely to be some sub-structuring, particularly for the Desert and Alpine ecotypes, and this is reflected in the significant *F*_IS_ values for these groups. The Mallee dingo samples, however, had the highest *F*_IS_ value and the lowest heterozygosity, suggesting significant inbreeding. The DIYABC-rf test indicated the best fit for the data was a single bottleneck (posterior probability of 0.49), with a reduction of 5,246 individuals as the Mallee population split from the Desert population (Supplementary Figure S3). However, this scenario was only marginally a better fit than a no bottleneck scenario, with both having a significant reduction in the effective population size (*N*_e_) for the Mallee population. The backcross, hybrid and dog samples all had the highest estimates of heterozygosity (Table 2).

**Table 1.** Pairwise *F*_ST_ between all dingo and dog populations with captive F1 hybrids (F1) and dingo backcrosses (Dingo BC).

	Alpine	Desert	Mallee	Dingo BC	F1
**Desert**	0.08				
**Mallee**	0.22	0.24			
**Dingo BC**	0.07	0.10	0.28		
**F1**	0.09	0.16	0.31	0.03	
**Dog**	0.27	0.34	0.42	0.17	0.08

**Table 2.** Descriptive summary statistics for each subpopulation sample genotyped in this study, including known captive hybrids and domestic dogs.

Subpopulation	*n*	*H*_O_	*H*_E_	u*H*_E_	*F*_IS_
Dingo—Alpine	248	0.20	0.24	0.24	0.14^1^
Dingo—Mallee	58	0.10	0.15	0.15	0.32^1^
Dingo—Desert	74	0.16	0.19	0.19	0.18^1^
Hybrid—F1 backcross	8	0.26	0.26	0.31	0.05^1^
Hybrid—F1	7	0.32	0.29	0.31	−0.03
Domestic dog breeds	39	0.27	0.29	0.30	0.10^1^

^1^Indicates significantly different from 0 after correction for multiple comparisons at the table-wide *α*ʹ = 0.05 level.

Clustering analysis on the Australian dataset showed clear differentiation between the Mallee group and the Alpine/Desert groups of dingoes (Figure 1C), while the domestic dogs were clearly differentiated from all dingo samples. A few samples were affected by missing data, particularly one Mallee sample (56% missing data; closer to the 0,0 location in Supplementary Figure S4). Interestingly, the known dingo-dog F1 hybrids and dingo-dog-dingo backcrosses grouped where expectations would predict. When removing the outlying Mallee population (which has high inbreeding and is the most isolated dingo group), we found a near perfect separation between domestic dogs, hybrids, backcrosses, and dingoes (Supplementary Figure S5). Clustering analysis on the global dataset shows a very similar pattern (Figure 1D), except that other canids (wolf, dhole, coyote, jackal, Andean fox) form their own separate differentiated group, all domestic breed dogs cluster together, with village dogs showing more variance and overlapping with some known F1 dingo-dog hybrids, while dingoes (Alpine, Desert, Mallee) cluster together with NGSD, highlighting the close relationship with NGSD.

**Figure 2.**
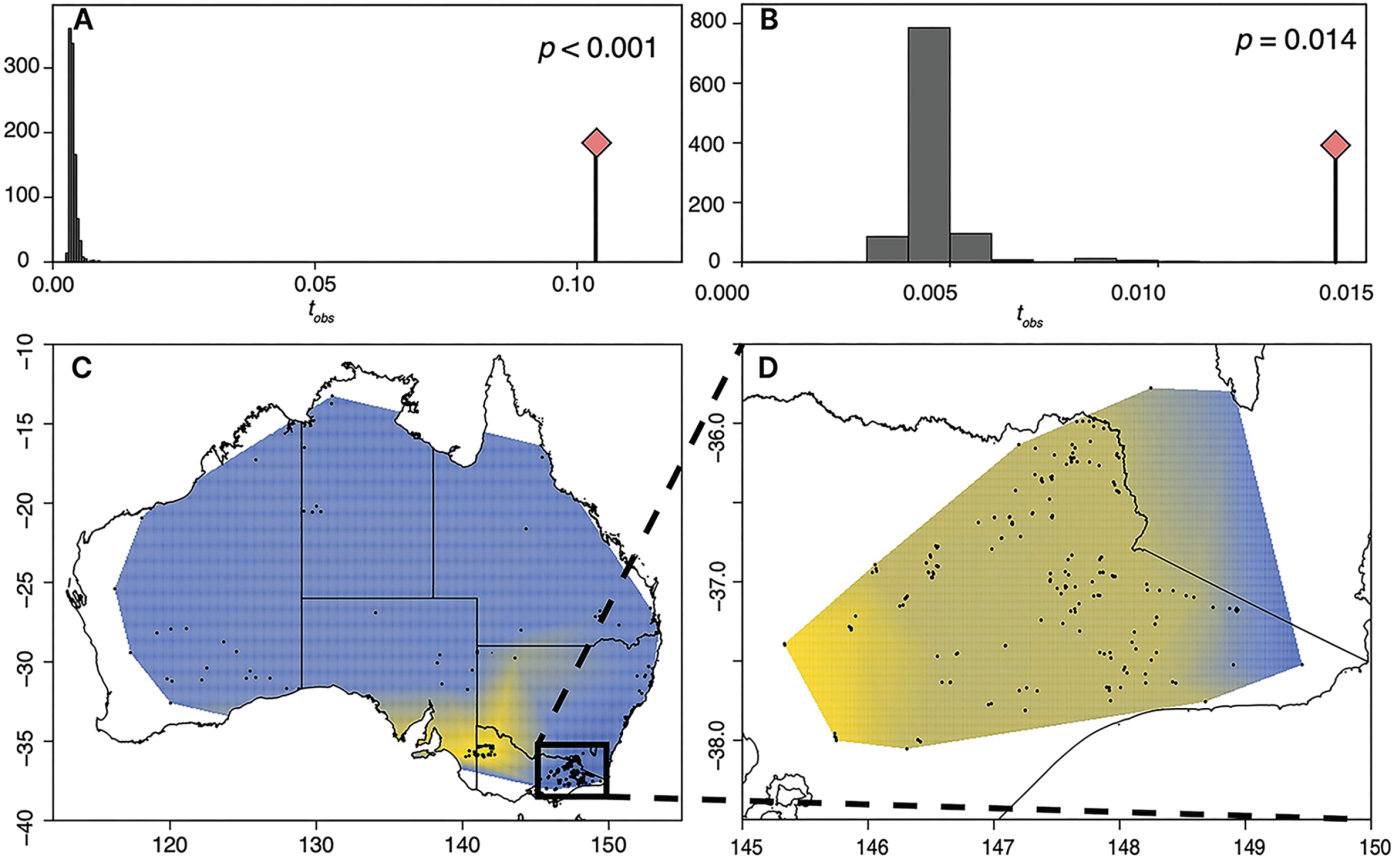
Isolation by distance (IBD) analysis for all dingo samples (*n* = 434) and for just the Alpine samples (*n* = 248). Significance through permutation for all dingoes (A) and just for Alpine dingoes (B), with a heatmap of Australia-wide patterns of spatial autocorrelation (C) and Alpine specific patterns of spatial autocorrelation (D). For (A) and (B), histogram includes the 1,000 permutations (expectations under a random model), the line with the red diamond is the observed statistic (*t*_*obs*_) from the data, and the *p*-value is the significant difference between *t*_*obs*_ and the expectations. For (C) and (D), yellow to blue represent a ramp of genetic difference due to spatial autocorrelation.

Individual STRUCTURE assignment analysis was performed on this data set to show that our data are similar to previous datasets. We found that the optimal *K*-value is four (Supplementary Figure S6a), which shows a clear distinction between domestic dogs (gray) and the three dingo groups (Supplementary Figure S6b), with relatively small Bayesian probability intervals of dog admixture across every individual, which also highlights the presence of dingo ancestry in several domestic dog samples (Supplementary Figure S6b). As expected, the known hybrids had proportions of dog and dingo that are reflective of being F1 (~50% dingo) and backcrossed (~75% dingo) individuals. There is a small proportion of potential admixture between dingo individuals and dogs in the Alpine population, and to a lesser degree in the Desert population (but not the Mallee population). This suggests that our dataset is no different to previous work. However, before identifying this as admixture, we explicitly test if this is due to sources other than admixture (see below in the dingo-dog admixture section). We then tested whether IBD is an explanatory variable in the genetic structure of dingoes. Using sPCA we found that there is a significant presence of IBD across Australia (Figure 2A and C). While this Australia-wide pattern appears to be driven by the Mallee population, we also found significant patterns of IBD within the Alpine group (Figure 2B and D).

Our results clearly suggest that structuring is present within the dingo group, a finding that contrasts with those from Stephens et al. (2015). To test whether that same structure is present within the Stephens et al. (2015) dataset, we reanalyzed their microsatellite data without using their “reference population” approach to see if the same structure was present in this extensive sample dataset. Indeed, we found clear patterns of IBD which question their original interpretations, with significant patterns of IBD among wild dingo samples (Supplementary Figure S7), and complete differentiation between dogs and dingoes using clustering analysis (Supplementary Figure S8). The different patterns of Australia-wide IBD between datasets are largely driven by presence (our SNP dataset) or absence (microsatellite dataset from Stephens et al. (2015)) of the highly inbred Mallee population.

### Dingo-dog admixture

**Figure 3.**
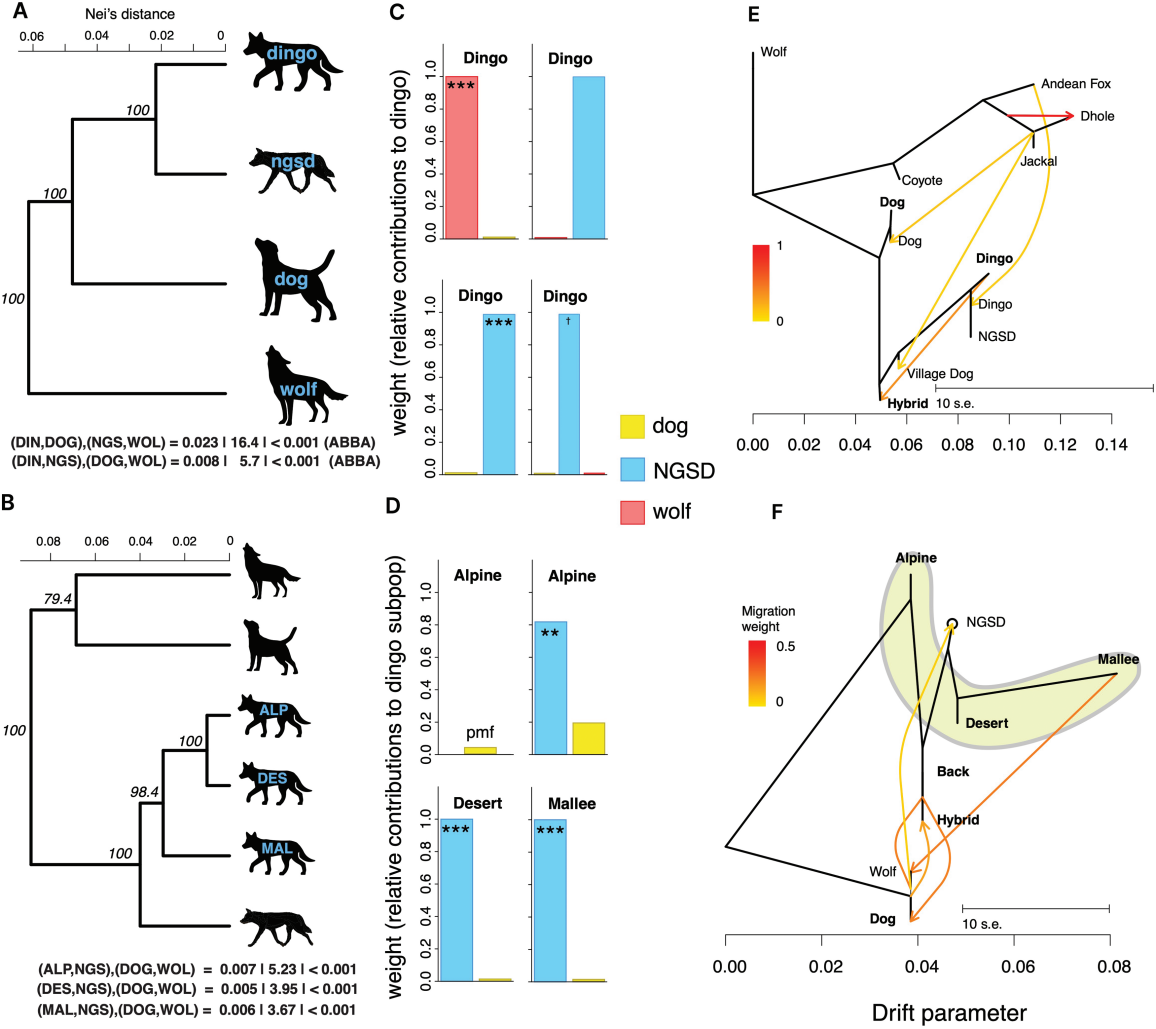
Evolution history and introgression between dingoes and other closely related canid groups. Phylogenies created using genetic distance (Nei’s distance) between canid groups and branch lengths are relative to one another and bootstrap supports are provided for each node. D-stat tests are provided between dingo (population 1; target), NGSD (population 2), domestic dog (population 3), and wolf (population 4; outgroup) (A). The model estimate, z-score, and *p*-value (D-stat estimate | z-score | *p*-value) are provided for each independent D-stat test. Then the dingo group was split into three separate populations to test for separate admixture events (B). The significantly positive z-scores indicate that the excess of ABBA topologies is significant for all genealogies (more admixture between NGSD and dogs than between dogs and dingoes). Relative genetic contributions to dingoes from each canid group were estimated for the dingo group (C) and each dingo population (D). Model fit is provided for each of the eight models in Supplementary Table S4. For each model (barplot) the target group (dingo group or population) is listed at the top of each barplot. Significant genetic contributions from specific donors (a single bar) are given asterisks (***<0.001; **<0.01; *<0.05; ^†^<0.1; pmf = no contribution due to poor model fit). If no * are present within a bar, then there were no significant contributions from that source group, meaning this genetic contribution is not significantly different to zero. We used a Treemix test to see if at least some admixture could be found between the dingo group and dogs (E), and between dingo populations (highlight with a yellow background) and dogs (F). We show a total of five possible admixture events for each independent analysis. In the Treemix figures, bold names are from our dataset, and other names are from the publicly available dataset.

We explicitly test for potential admixture between dingoes and their canid relatives using D-stat (ABBA-BABA) derived tests and Treemix to see if domestic dogs shared genetic variation with dingoes compared to other canids. We first created a phylogenetic tree based on genetic distance (Figure 3A and B). Using the tree, we set up the D-stat test (using Admixtools) to compare the four canid groups (dingo, NGSD, domestic dog, and wolf) to see if there was greater gene flow between dingoes and dogs than expected. The D-stat is significantly positive (*p* < 0.001) regardless of where we placed NGSD and domestic dogs (Supplementary Table S3), showing that there has been gene flow between NGSD and domestic dogs (Figure 3A). This ABBA pattern suggests that there is more admixture between NGSD and domestic dogs compared to the possible admixture between dingoes and domestic dogs. We then used admixture weighting analysis (qpAdm) to determine the amount of genetic variation in the target (dingo) group from NGSD, dog, and wolf groups and found that genetic variation in dingoes is only significantly shared with NGSD in two of the tests (Figure 3C). All qpAdm models were good fits with *p* values > 0.05 (see Figure 3C and D, above each barplot; Supplementary Table S4). Here, NGSD are the only significant contribution to the dingo group in the two-lineage models, but this signal is mostly lost when three lineages are used, although there was a marginally significant contribution from NGSD (*p* = 0.06). It should be noted that these barplots must be equal to one, regardless of significance (i.e., nonsignificant bars are not different to zero). However, we thought that each dingo population could have independent admixture events with domestic dogs and therefore ran the D-stat test for the three populations (Alpine, Desert, Mallee) to determine if any dingo population had a significant amount of domestic dog introgressed genetic variation. We found that there is more admixture between NGSD and domestic dogs compared to the admixture between each dingo population and domestic dogs (Figure 3B). We also performed the admixture weighting analysis on each dingo population independently to see if NGSD or domestic dogs significantly shared genetic variation with each dingo population, and we found that each population had a significant amount of genetic variation from NGSD (Figure 3D). The Alpine population had a higher nonsignificant relative contribution (0.20) from domestic dogs, so we ran the same analysis with the Alpine population as the target and domestic dogs as the only source population and found a poor model fit overall because the estimated and fitted model significantly differed (*p* < 0.001; Figure 3D; Supplementary Table S4).

Finally, we used Treemix to test for ancient admixture between domestic dogs and dingoes. With a possible five connections (which was the optimal number of migration events for both groupings with the smallest residuals; standard error plots in Supplementary Figure S9), we found that there was no connection between dingoes and domestic dogs (Figure 3E) when using all canid groups in our dataset. Even when focusing on the focal canid groups in our study (dingo, dog, NGSD, and wolf), three dingo populations and known hybrids, we found no admixture connection between dingo populations and dogs (Figure 3F). However, there was a connection between the Mallee population and the wolf group, and a clear connection between hybrids (known captive F1 and backcrosses) and domestic dogs.

## Discussion

The results of our genomic SNP analyses of 434 Australian canid samples indicate significant population structure across the distribution of free ranging canids (dingoes) in Australia. The three broadly classified dingo populations (Alpine, Desert, and Mallee) had a strong IBD genetic pattern both globally (across Australia) and regionally within the Alpine dingo population. There is likely to be further structure in the Desert population, as indicated by the significant excess of homozygotes in the SNP data and supported through reanalysis of past microsatellite data, although further sampling would be required in our dataset to disentangle this pattern. Our results are consistent with the recent genomic SNP analysis in Cairns et al. (2023), although they sampled more extensively on the east coast of Australia in New South Wales and Queensland, identifying an additional population in this region. Similarly, our results are consistent with a recent ancient DNA genomic study of dingoes (Souilmi et al., 2024), which highlighted that the population structure is historic and likely related to multiple introductions of dingoes to Australia. In all our analyses, including with the global dataset, domestic dogs are highly differentiated from the three dingo populations. The global dataset also showed the NGSD is the closest canid relative and only genetic contributor (from canid groups in our dataset) to the Australian dingo, consistent with previous analyses indicating their likely origin from the New Guinea region (Souilmi et al., 2024; Surbakti et al., 2020).

We found no evidence of recent hybridization between domestic dogs and dingoes across Australia in our dataset. Our direct tests of hybridization did not find evidence of hybridization between dingoes and domestic dogs, regardless of whether we treat the dingo populations independently or as one group. Similarly, the population structuring from significant IBD in dingoes argues against previous work that suggested widespread and pervasive hybridization with domestic dogs (Stephens et al., 2015), as pervasive hybridization should lead to the breakdown of this genetic pattern. If hybridization is occurring, it occurs relatively rarely as is shown in the recent study comparing ancient dingo genomes to modern ones (Souilmi et al., 2024). Our work has focused on population level allele frequency changes across groups and suggests that any admixture is not being significantly reflected at the population level, although we cannot discount some very low levels of introgression. The suite of tests we performed are not perfect and rely on other canid data and currently known evolutionary history to disentangle admixture from the complex common ancestry between dingoes and dogs. Regardless, we were able to accurately characterize known captive F1 hybrids and backcrosses, highlighting that clustering methods were sensitive to recent hybridization events. Our data therefore suggests no effective introgression from domestic dogs to dingoes since dingoes split from the NGSD approximately 5,000–8,300 years ago (Ballard & Wilson, 2019; Souilmi et al., 2024; Zhang et al., 2020).

These results are in stark contrast to previous genetic analyses on dingoes using microsatellite loci (Stephens et al., 2015), where hybridization was found throughout their distribution in Australia, with approximately 54% of 3,637 dingoes identified as hybrids. In their study, the highest frequency of hybridization occurred in southeastern Australia, where 99% of individuals analyzed were identified as hybrids. A more recent analysis using genomic SNP loci (Cairns et al., 2023), however, found much lower levels of hybridization across their distribution (~30% of 307 samples), with only 13% of animals from southeastern Australia (Victoria) identified as dingo backcrosses. So, how do we reconcile our results with these studies?

The methods used to identify hybrids in Stephens et al. (2015) assumed no population structure in dingoes across Australia and used predetermined reference populations of “pure” dingoes and domestic dogs to identify hybrid individuals. The reference “pure” dingo population contained predominately individuals from Western and Central Australia, where it was assumed hybridization was likely to be lowest. Hybrids were then identified using a simple *q*-value estimation procedure implemented in the program STRUCTURE, where the proportion of an individual was estimated that belonged to each reference population. The use of reference populations in analyses combined with an assumption of no population structure biased their results and interpretation, resulting in any dingo sample that is different to the “pure” dingo reference artificially lying between the two references. This led to the erroneous conclusion that 99% of the structured wild canid samples in southeastern Australia are hybrids. Our reanalysis of their dataset illustrates this point, where we find the same genetic patterns of structure and IBD as that found in our SNP dataset (Supplementary Figures 7 and 8).

To be consistent with the previous research, the same *q*-value approach was implemented in Cairns et al. (2023) with genomic SNP data, although in this case no reference populations were used in the model, 95% confidence interval *q*-values were estimated in fastStructure and individuals were assigned to ancestry categories with only three categories found in wild dingo populations (>99% dingo, >93% dingo, and 55%–93% dingo, with the latter two categories labeled historic dingo backcrosses and recent dingo backcrosses, respectively). Importantly, the *q*-value methods employed by both studies can perform poorly when biological conditions deviate from the inference model (e.g., recent bottlenecks, lethal control) and overlook the evolutionary history of dingoes and the likely shared ancestry with early dog lineages dating back to their split from wolves (Field et al., 2022). Under a simple allopatric speciation model, drift alone requires 9–12 *N*_e_ generations to make incipient species reciprocally monophyletic at more than 95% of loci (Hudson & Coyne, 2002), which for relatively recently diverged populations will likely result in considerable shared genetic variation (Lopes et al., 2021). Our analyses, therefore, suggest that this shared ancestry derived from admixture analyses such as STRUCTURE has resulted from ILS, where ancestral polymorphisms are retained and discordant to the species tree (Degnan & Rosenberg, 2009), rather than recent admixture between dingoes and domestic dogs. ILS has been commonly found in marsupials (Nilsson et al., 2018), marine mammals (Lopes et al., 2021), birds (Jarvis et al., 2014), insects (Pollard et al., 2006), and in recently diverged species such as neotropical birds (Suh et al., 2015), bats (Korstian et al., 2022) and Hawaiian tropical plants (Kleinkopf et al., 2019). In canids, ILS has also been hypothesized as an explanation for shared ancestry in red wolves from other canids (coyotes and gray wolves), although recent introgression has also been identified (National Academies of Sciences & Engineering, 2019). ILS can look like recent admixture when using clustering algorithms, whereas our direct tests using ABBA/BABA analyses and Treemix highlight that the admixture with domestic dogs detected previously (Cairns et al., 2023; Stephens et al., 2015) is unlikely to be recent. In fact, we show that the derived allele is shared between NGSD and domestic dogs at a significantly higher frequency than between dingoes and domestic dogs.

We speculate that social structure and dingo behavior are significant pre-zygotic barriers preventing introgression with domestic dogs in Australia. Similar mechanisms operate in other canid groups, such as social behaviors preventing hybridization between gray wolves and coyotes (Merkle et al., 2009) or size assortative mate choice limiting hybridisation between reintroduced red wolves and coyotes (Hinton et al., 2018). In these instances, however, harvesting can cause significant ecological disruption to the social dynamics of wolves, leading to hybridization with coyotes in areas where coyotes are abundant. Similar issues have been observed in Europe between gray wolves and dogs, where male biased introgression occurs from dogs into wolf populations and lethal control of wolves has resulted in increased hybridization (Pilot et al., 2018). Lethal control is used broadly in Australia, particularly in eastern Australia, where there is significant conflict between dingoes and livestock producers. In Victoria, livestock losses only represented 0.86% of the $13M (AUD) cost directly related to dingo management, with the rest being spent on control efforts or incentives between 2017 and 2018 (Boronyak & Jacobs, 2023). Consequently, these large-scale lethal control programs may have major biological and evolutionary ramifications in future dingo-dog relationships; it is possible, even likely, that continued lethal control may break down behavioral barriers leading to future introgression, as seen in wolf-dog dynamics in Europe (Pilot et al., 2018).

In Australia, the dingo has a complicated public policy history where state-specific government policy can both protect and persecute the dingo. The dingo fence, a ~5,600 km structure built to protect livestock in southeastern Australia in the 1950’s (Figure 1A), limits movement of dingoes with lethal control heavily practiced east of the fence in South Australia, Queensland, and New South Wales where dingoes are called wild dogs. In the state of Victoria (south-east of the dingo fence), however, the dingo is considered a threatened species (Victorian Government, 2023a), but wild dogs and dingo-dog hybrids are considered vermin and lethal control is encouraged by government (Victorian Government, 2023b). Our results highlight that there is not a measurable amount of domestic dog variation detected in Victorian dingoes, and therefore lethal management is currently leading to the culling of a legally protected threatened species. The impact of this culling on population size in dingoes is currently unknown in the Alpine population, but clearly it has had severe impacts in the Mallee population. The Mallee dingo population shows evidence of significant inbreeding and very low estimates of genetic diversity compared with the Alpine and Desert populations. This population is largely restricted to two small parks in Victoria on the South Australian border. Despite its significance to First Nations People, active lethal management (baiting, trapping, and shooting) occurs around these parks as part of both state governments wild dog management programs to prevent livestock loss.

Our results indicate that the Mallee population is very small, completely isolated, and at threat of imminent local extinction. From a conservation perspective, active genetic management is likely to be necessary to ensure persistence of this legally protected population. This should entail the introduction of individuals from another dingo population to reduce the effects of genetic drift and inbreeding and increase genetic variation and adaptive potential (Weeks et al., 2011). The results indicate that the Desert population is more closely related to the Mallee population, but the Alpine population contains more genetic variation. Introducing individuals from either would likely provide a fitness benefit (Hoffmann et al., 2021; Weeks et al., 2017), but the population will likely be lost through mortality (i.e., “extinction vortex”) in the near future without reducing lethal management.

The dingo has undergone over 5,000 years of independent evolution without the presence of another canid in Australia, creating a unique lineage that is differentiated from other canids and on an early pathway towards speciation. While dingoes, NGSD and domestic dogs have a shared ancestral history, significant pre-zygotic barriers are likely preventing hybridization between dingoes and domestic dogs, and allowing several genetically differentiated dingo populations to persist in Australia. However, as seen in other parts of the world, lethal control and continued habitat destruction are the main threats that could break down the ongoing evolutionary pathway to speciation by increasing the likelihood of hybridization and introgression with domestic dogs. Whether the dingo continues along the speciation trajectory likely depends heavily on public policy in Australia and the continued lethal management in south-eastern Australia. Therefore, it is important to adopt and promote coexistence pathways that protect productive rangeland systems and the dingoes’ evolutionary future as mainland Australia’s largest apex predator, which comes with numerous ecological benefits (Fisher et al., 2021; Letnic et al., 2012).

## Supplementary material

Supplementary material is available online at *Evolution Letters*.

qrae057_suppl_Supplementary_Tables_S1-S4_Figures_S1-S9

## Data Availability

The genomic SNP data generated in this study for the 434 individuals (VCF files) are available on the Figshare repository (https://doi.org/10.6084/m9.figshare.27022555.v1), along with code for analyses.
