# Supplementary material for: Genetic structure and common ancestry expose the dingo-dog hybrid myth
Source: Evol Lett. 2024 Oct 19;9(1):1–12. doi: 10.1093/evlett/qrae057 (PMC11790216; doi:10.1093/evlett/qrae057)
Supplement: qrae057_suppl_Supplementary_Tables_S1-S4_Figures_S1-S9 [file qrae057_suppl_supplementary_tables_s1-s4_figures_s1-s9.zip › Weeks et al 2024_1October2024_SUPPINFO.pdf]

Supplementary Information

Genetic structure and common ancestry expose the dingo-dog hybrid myth

Andrew R. Weeks[1,2], Peter Kriesner[1], Nenad Bartonicek[1], Anthony van Rooyen[1], Kylie M. Cairns[3], Collin W. Ahrens[1]

[1]Cesar Australia, Brunswick, Victoria, Australia

[2]School of BioSciences, The University of Melbourne, Parkville, Victoria Australia

[3]Evolutionary & Ecology Research Centre, School of Biological, Earth and Environmental Sciences, University of New South Wales, Sydney, New South Wales

Supplementary file	Title	Page
Table S1	Australian dataset metadata	2
Table S2	Global dataset metadata	2
Table S3	qpdstat results	2
Table S4	qpAdm results	3
Figure S1	PCA dingo aligned SNP dataset	4
Figure S2	PCA dingo aligned SNP dataset without Mallee population	5
Figure S3	DIYABC-rf results	6
Figure S4	PCA with missing data informed points	7
Figure S5	PCA dog aligned SNP dataset without Mallee population	8
Figure S6	STRUCTURE results SNP dataset	9
Figure S7	Isolation by distance results from microsatellite data	10
Figure S8	DAPC from microsatellite data	11
Figure S9	Treemix analysis residuals	12

Supplementary Table S1. Metadata for samples included in the Australian dataset. Available as a separate csv file.

Supplementary Table S2. Metadata for samples included from Plassais *et al.*, (2019). Available as a separate csv file.

Supplementary Table S3. Results for the qpdstat models. A positive z score indicates more ABBA topologies than BABA topologies. The top two rows are the Dstat for all of the dingo populations in one group, and the three rows in grey are the Dstat results for each dingo population.

**pop1**	**pop2**	**pop3**	**pop4**	**model estimate**	**se**	**z score**	***p* value**
dingo	dog	NGSD	wolf_public	0.0239	0.0015	16.4	< 0.001
dingo	NGSD	dog	wolf_public	0.0079	0.0014	5.7	< 0.001
Alpine	NGSD	dog	wolf_public	0.0065	0.0012	5.23	< 0.001
Mallee	NGSD	dog	wolf_public	0.0055	0.0015	3.67	< 0.001
Desert	NGSD	dog	wolf_public	0.0045	0.0011	3.95	< 0.001

Supplementary Table S4. Results for all of the qpAdm models in the manuscript. The reference populations for the first eight models included the following groups from the public database: Jackal, Dhole, Andean Fox, and Coyote. However, the reference group with a * included the NGSD group. The chi square metric tests if the estimated and fitted D-statistics significantly differed, where non-significance (p – model fit > 0.05) indicates that the model fits. The constrained weights are provided with z-scores within parentheses. The top four rows are the qpadm results for all of the dingo populations combined into one group, and the three rows in grey are the results separated for each dingo population.

**target**	**source**	**chisq**	**p - model fit**	**weight - dog**	**weight - wolf**	**weight - NGSD**
dingo	wolf,dog	0.78	0.68	< 0.001 (6.38e-22)	1 (9.42e+12)	NA
dingo	wolf,ngsd	2.29	0.32	NA	< 0.001 (1.60e-17)	1 (0.206)
dingo	ngsd,dog	3.20	0.20	< 0.001 (1.41e-17)	NA	1 (6.04)
dingo	wolf,ngsd,dog	3.18	0.20	0.02 (0.036)	0.04 (1.10)	0.98 (1.55)
Alpine*	dog	408.0	< 0.0001	–	–	–
alpine	ngsd,dog	1.29	0.53	0.201 (0.63)	NA	0.80 (2.48)
mallee	ngsd,dog	0.03	0.99	< 0.001 (7.48e- 2)	NA	1 (1.00e+13)
desert	ngsd,dog	1.78	0.41	< 0.001 (6.01e- 2)	NA	1 (1.00e+13)

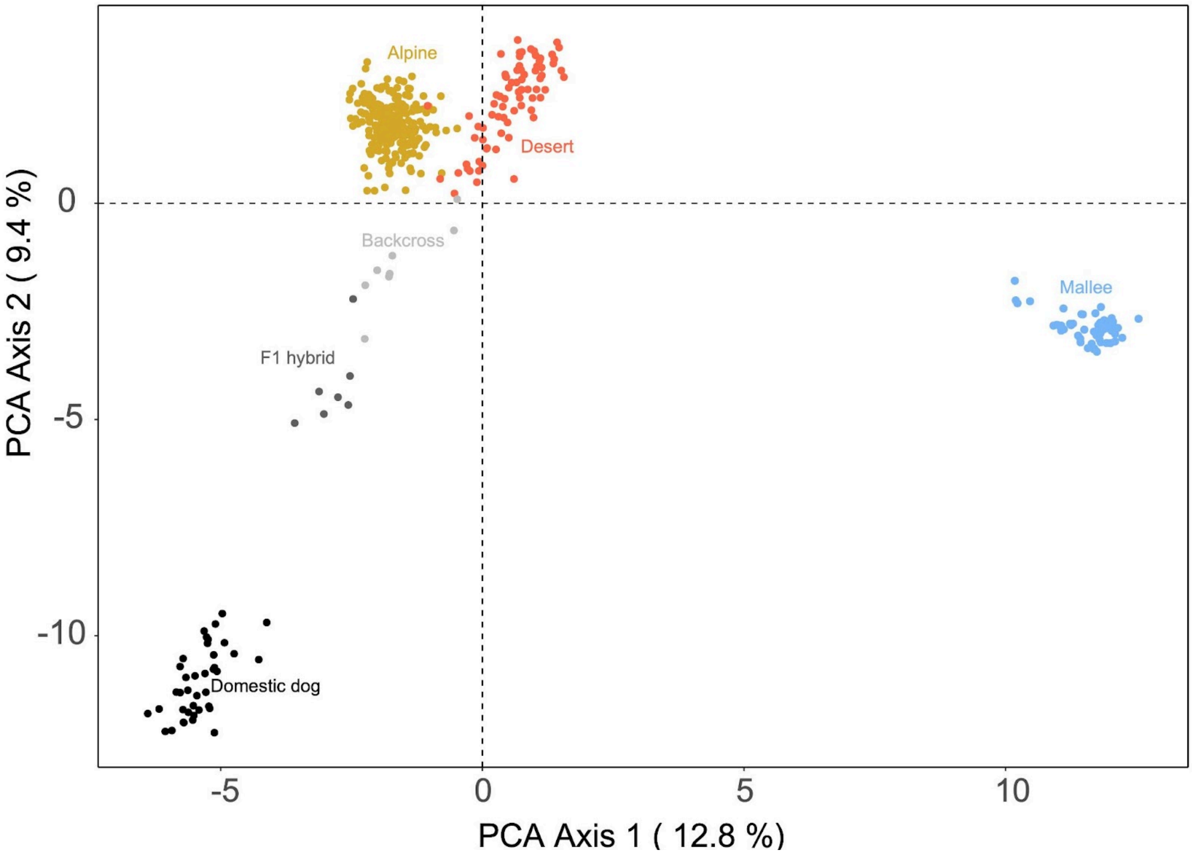

Supplementary Figure S1. PCA using the SNP dataframe that was aligned to the Dingo Genome.

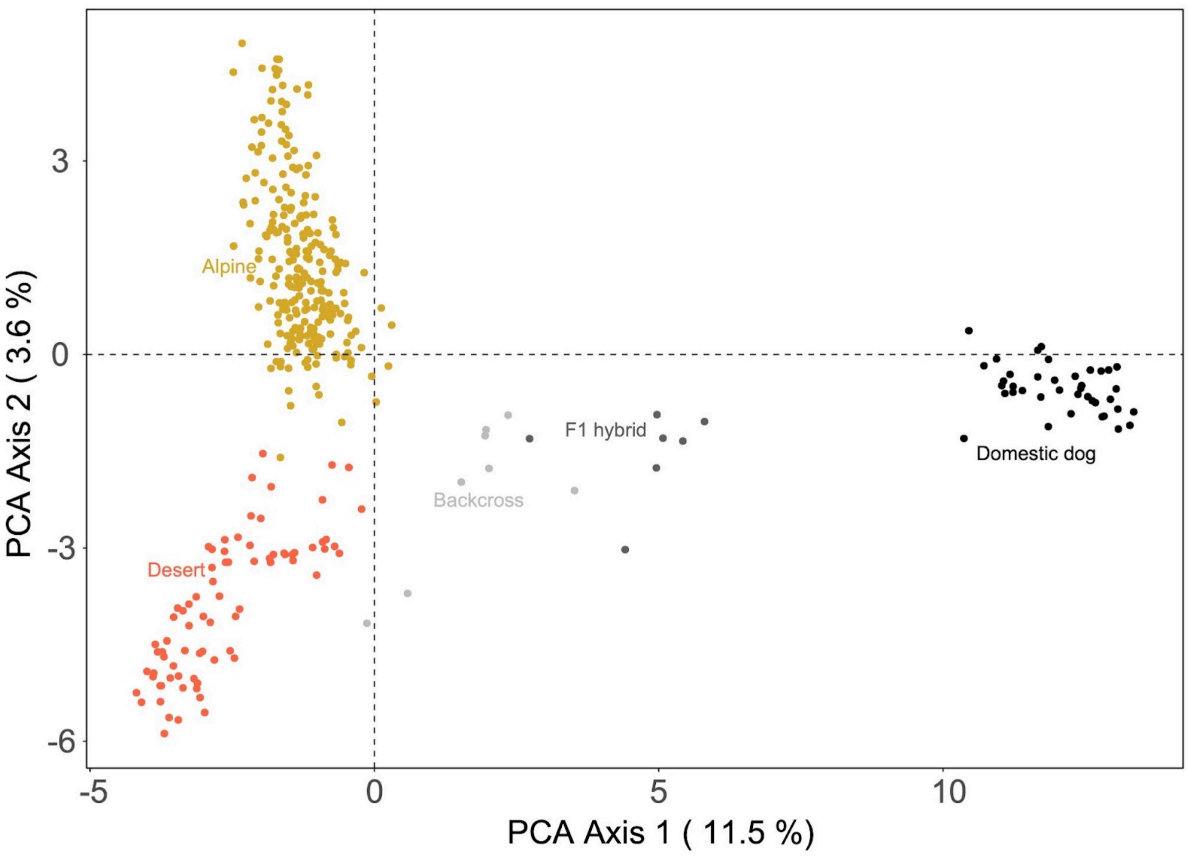

Supplementary Figure S2. PCA using the SNP dataframe that was aligned to the Dingo Genome without the Mallee population.

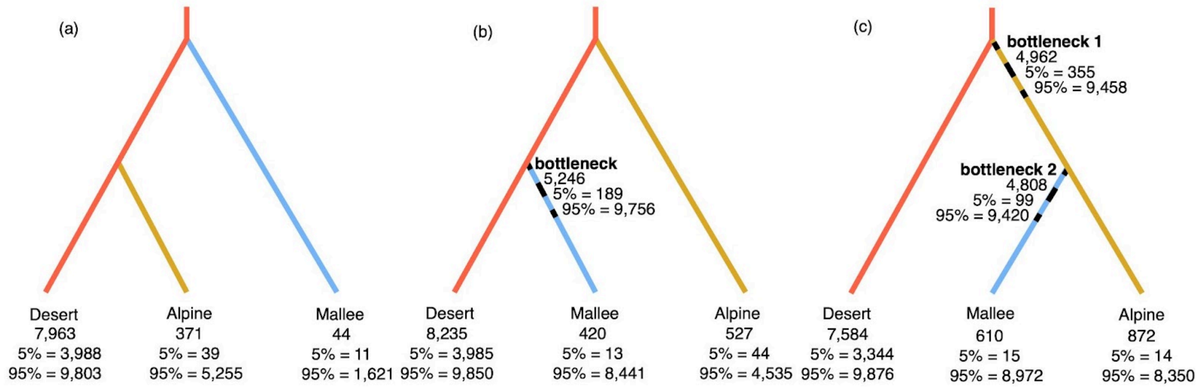

Supplementary Figure S3. A test for a population bottleneck for the Mallee population. Of a possible 27 different scenarios, the top three models are shown above for no bottlenecks (a), one bottleneck (b), and two bottlenecks (c). The scenario with one bottleneck had the best performance but was followed closely by the scenario with no bottlenecks. Under each population name, we provide the effective population size estimate for each population and the 5% and 95% confidence intervals. Bottleneck information is provided next to the dashed line, which is the decline in effective population size and its confidence intervals.

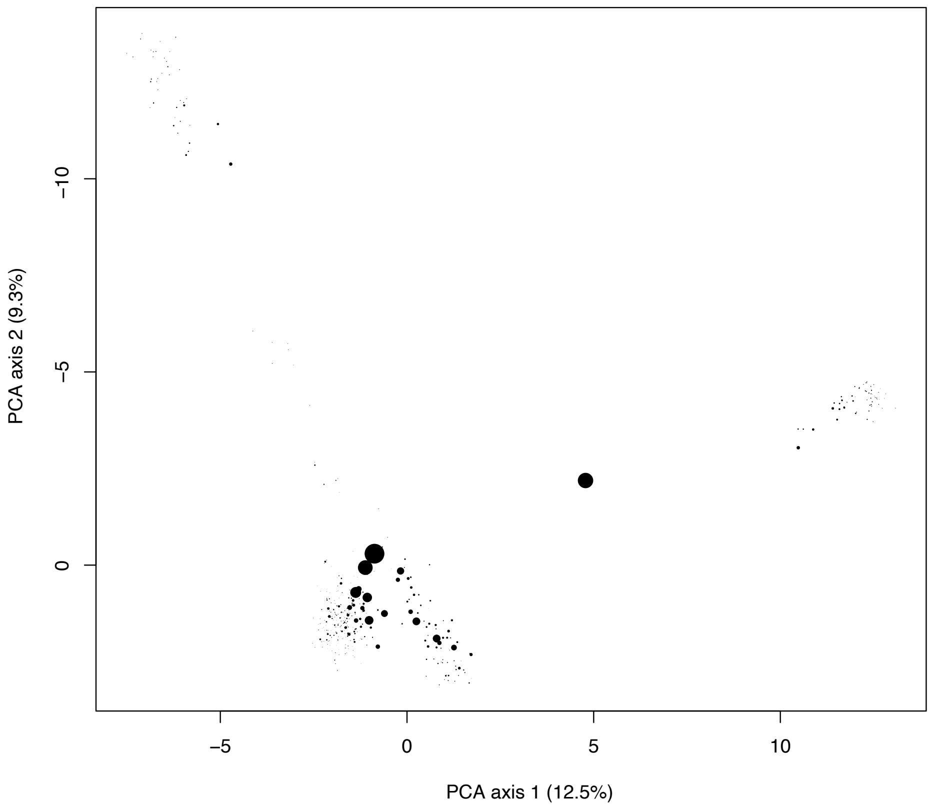

Supplementary Figure S4. PCA with missing data as size of circle. In total, three samples have inflated missing data (76%, 59% and 57%). Samples with missing data were closer to the 0,0 location of the plot.

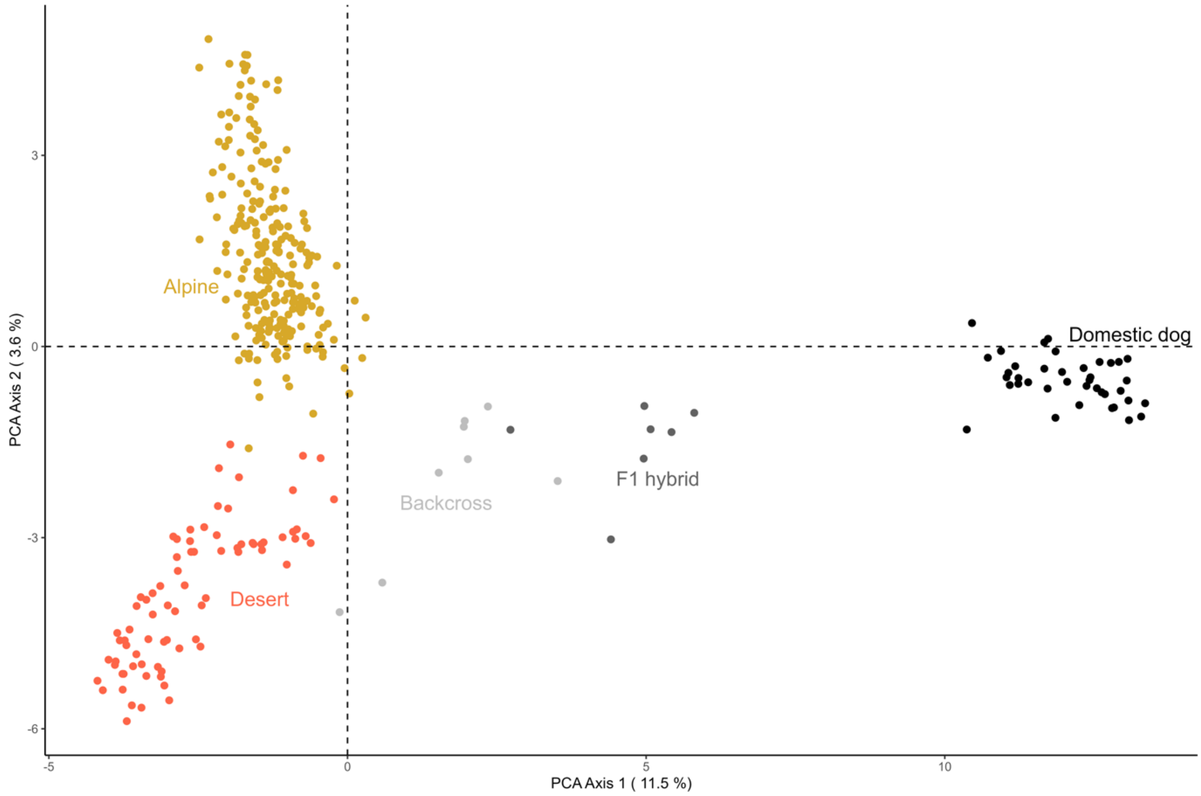

Supplementary Figure S5. PCA on individuals genotyped aligned to the dog genome in this study with individuals from the Mallee dingo population removed (n = 376).

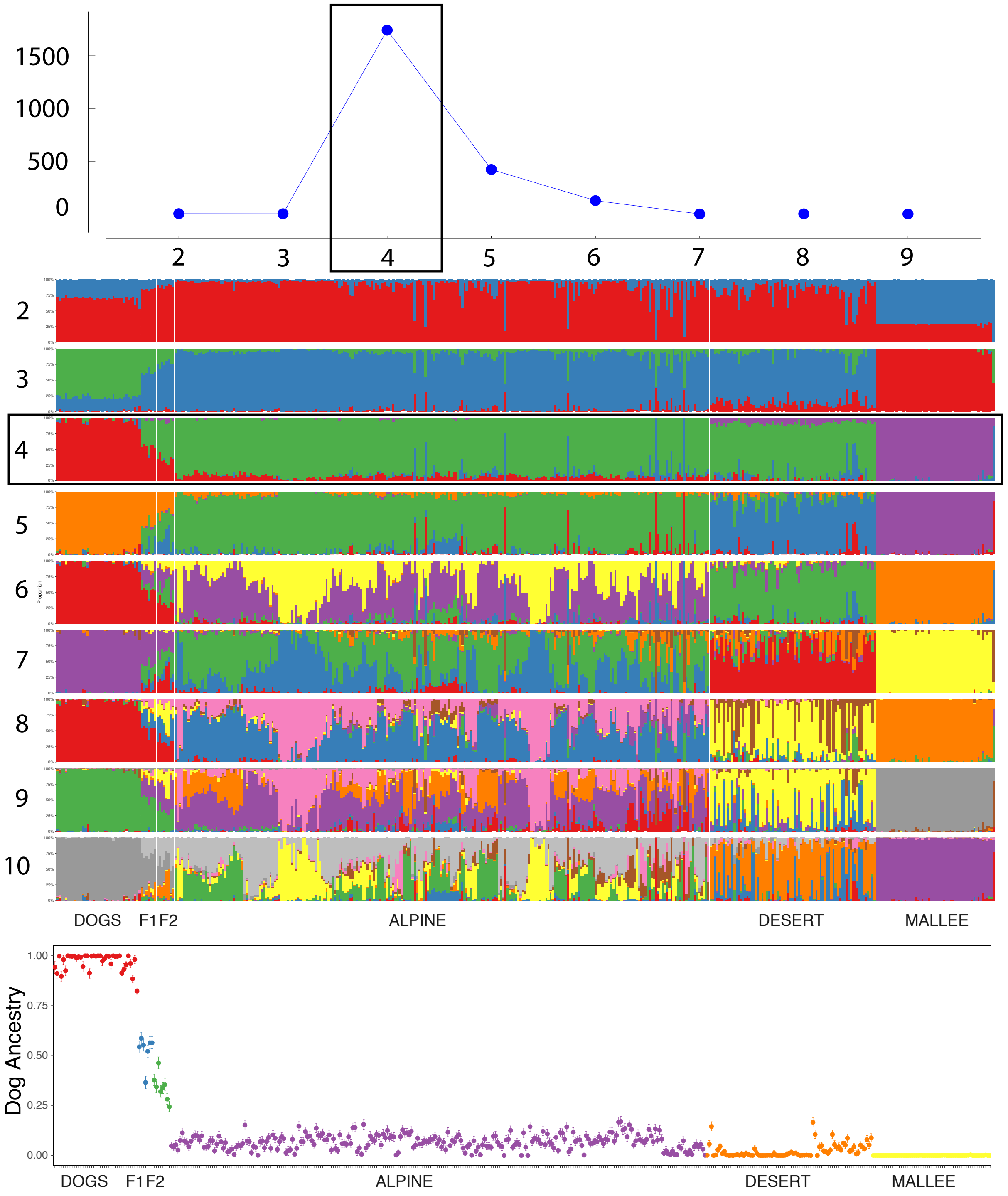

Supplementary Figure S6. Full STRUCTURE results for K = 1 to 10 (10 reps per K), with the change in ΔK indicating K = 4 is the optimal number of populations. All individuals genotyped in this study are included in analyses (n = 434). Probability intervals of the dog ancestry for all individuals.

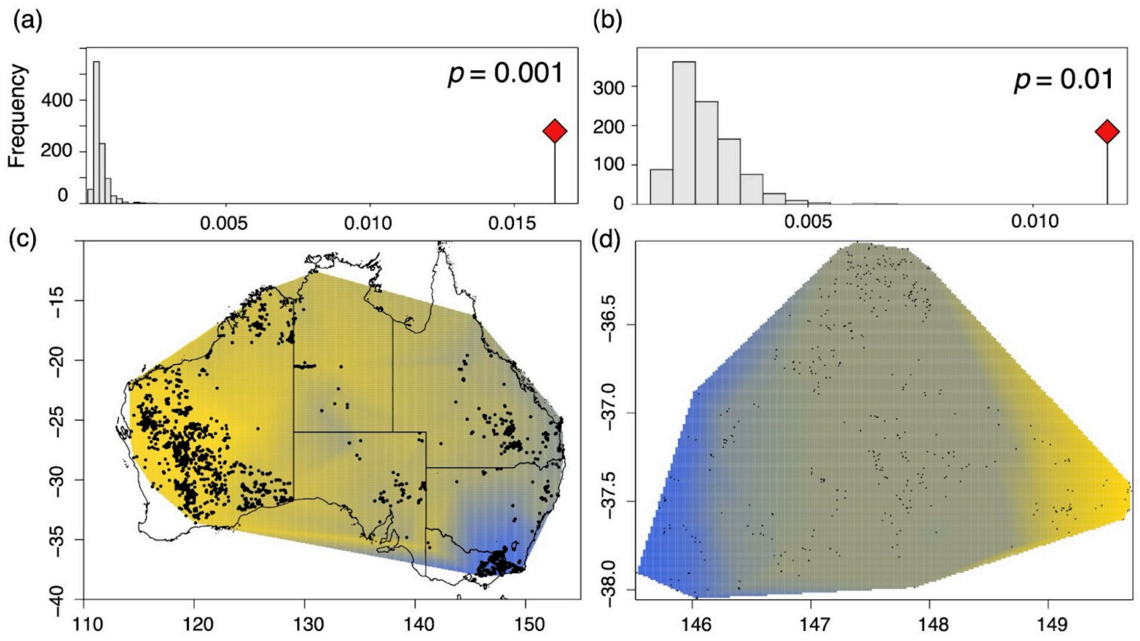

Supplementary Figure S7. Isolation by distance analysis on the microsatellite data from Stephens et al. (2015) (obtained from the dryad repository under doi:10.5061/dryad.2rd32). This figure is directly comparable to Figure 2 in the main text. Significance through permutation for all dingoes (a) and just for Alpine dingoes (b), with a heatmap of Australia-wide patterns of spatial autocorrelation (c) and Alpine specific patterns of spatial autocorrelation (d). For (a) and (b), histogram is the 1000 permutations and the line with the red diamond is the observed statistic (t_{obs}, difference between arithmetic means) from the data. For (c) and (d), colours represent a ramp of genetic difference due to spatial autocorrelation.

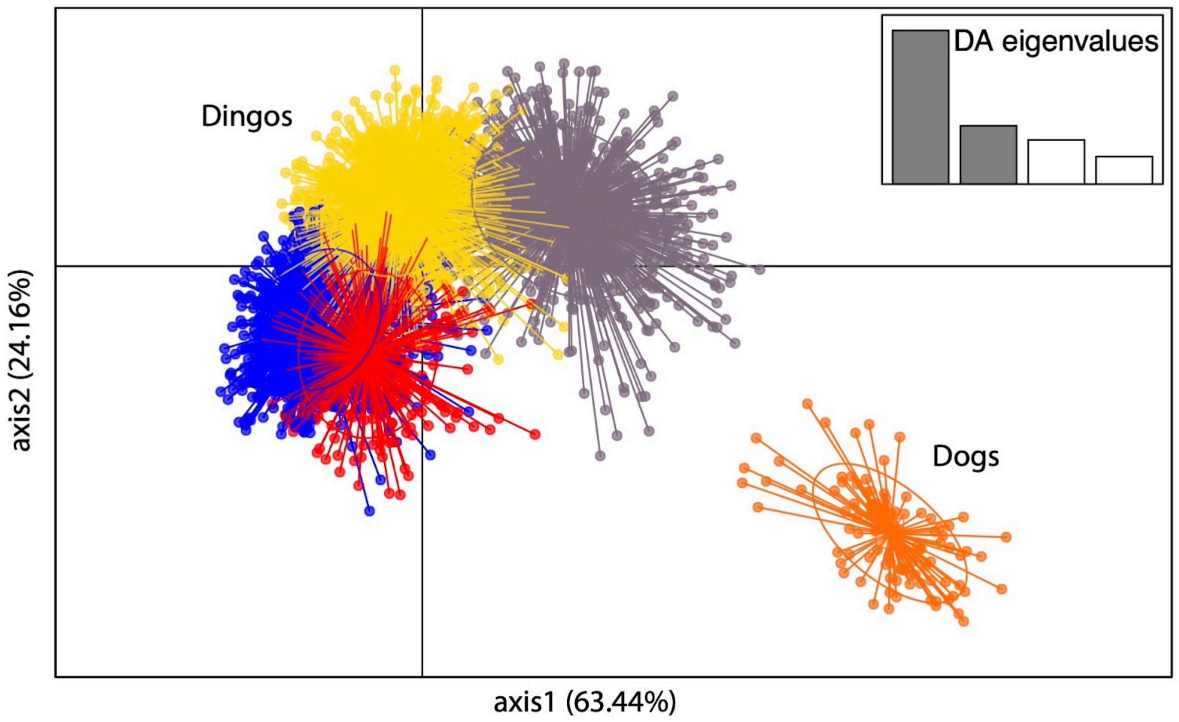

Supplementary Figure S8. DAPC on the microsatellite data from Stephens et al. (2015) (obtained from the dryad repository under doi:10.5061/dryad.2rd32). The optimal value of K = 5 clusters and each K-value corresponds to an ancestral genetic cluster. But generally, the grey is the southeastern Australian samples, yellow is the northeastern samples, while the blue and red represent the central and western samples (map of samples in Figure S7).

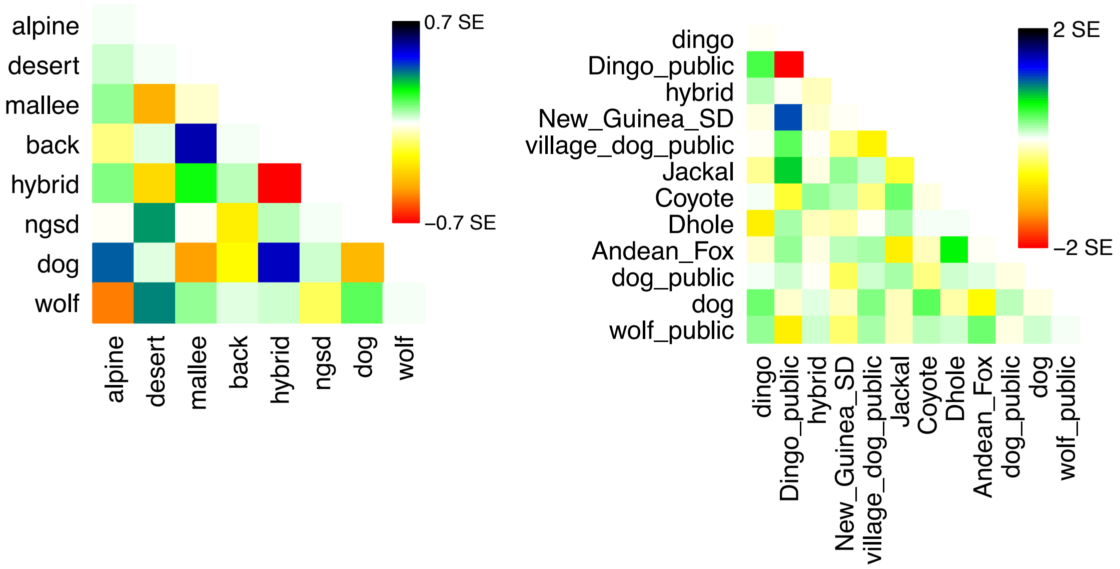

Figure S9. Residuals for the Treemix analyses. Both support five total connections as being the optimum for each analysis.
